# Transcriptome Analysis of NPFR Neurons Reveals a Connection Between Proteome Diversity and Social Behavior

**DOI:** 10.3389/fnbeh.2021.628662

**Published:** 2021-03-31

**Authors:** Julia Ryvkin, Assa Bentzur, Anat Shmueli, Miriam Tannenbaum, Omri Shallom, Shiran Dokarker, Jennifer I. C. Benichou, Mali Levi, Galit Shohat-Ophir

**Affiliations:** ^1^The Mina and Everard Goodman Faculty of Life Sciences, Bar-Ilan University, Ramat Gan, Israel; ^2^The Leslie and Susan Gonda Multidisciplinary Brain Research Center, Bar-Ilan University, Ramat Gan, Israel

**Keywords:** *Drosophila melanogaster*, behavior, motivation, reward, social interaction

## Abstract

Social behaviors are mediated by the activity of highly complex neuronal networks, the function of which is shaped by their transcriptomic and proteomic content. Contemporary advances in neurogenetics, genomics, and tools for automated behavior analysis make it possible to functionally connect the transcriptome profile of candidate neurons to their role in regulating behavior. In this study we used *Drosophila melanogaster* to explore the molecular signature of neurons expressing receptor for neuropeptide F (NPF), the fly homolog of neuropeptide Y (NPY). By comparing the transcription profile of NPFR neurons to those of nine other populations of neurons, we discovered that NPFR neurons exhibit a unique transcriptome, enriched with receptors for various neuropeptides and neuromodulators, as well as with genes known to regulate behavioral processes, such as learning and memory. By manipulating RNA editing and protein ubiquitination programs specifically in NPFR neurons, we demonstrate that the proper expression of their unique transcriptome and proteome is required to suppress male courtship and certain features of social group interaction. Our results highlight the importance of transcriptome and proteome diversity in the regulation of complex behaviors and pave the path for future dissection of the spatiotemporal regulation of genes within highly complex tissues, such as the brain.

## Introduction

Behavior is the result of an orchestrated neuronal activity, where a complex collection of cell types assembled into circuits process external and internal information into a consistent motor output that ultimately promotes survival and reproduction (Bargmann and Marder, 2013; Anderson, 2016; Chen and Hong, 2018; Datta et al., 2019). The immense complexity and heterogeneity of the nervous system results from molecular programs that dictate the range of expressed proteins, including their localization and function, giving rise to cell populations with diverse anatomy, physiology, connectivity, and functional roles (Cabrera, 1992; Franco and Müller, 2013; Mo et al., 2015; Chen et al., 2017; Gray and Spiegel, 2019; Mickelsen et al., 2019; Sapiro et al., 2019; Winnubst et al., 2019; Xu et al., 2020). This diversity poses a challenge when trying to functionally associate neurons to particular behaviors but can be resolved by genetically dividing the brain into discrete cell types and subsequently study their anatomy, connectivity, molecular architecture and physiology (Henry et al., 2012; Croset et al., 2018; Agrawal et al., 2019; Shih et al., 2019; Davis et al., 2020). Recent advances in targeting increasingly smaller sub populations of neurons, together with tools to manipulate their activity, make it possible to connect the function of neurons to their identity, thus facilitating greater understanding of the molecular underpinning of brain development and mechanisms that regulate complex behaviors (Venken et al., 2011; Yizhar, 2012; Waddell et al., 2015; Abruzzi et al., 2017; Anpilov et al., 2020). This can be useful when studying the function of neurons that control complex behaviors, particularly those that are regulated by motivation such as foraging, food and water consumption, mating and various forms of social interactions (Goodson and Bass, 2001; Desai et al., 2013; Arias-Carrión et al., 2014; Anderson, 2016; LeGates et al., 2018; Parker et al., 2019; Senapati et al., 2019; Sternson, 2020).

The fruit fly *Drosophila melanogaster* is a useful model organism for investigating the genetic underpinnings of motivational behaviors, owing to variety of tools for neuro-genetic manipulations, together with the fact that flies exhibit several forms of behaviors that are shaped by motivation (Wu et al., 2003; Certel et al., 2007; Krashes et al., 2009; Aso et al., 2014; Perisse et al., 2016; Bentzur et al., 2018; Pu et al., 2018; Zer-Krispil et al., 2018; Zhang et al., 2018; Senapati et al., 2019; Wilinski et al., 2019; Thornquist et al., 2020). One of the systems that encodes internal states and dictates motivational drives, and consequently, behavioral choices in *Drosophila* is the Neuropeptide F/Neuropeptide F receptor (NPF/R) (Wen et al., 2005; Wu et al., 2005; Lingo et al., 2007; Xu et al., 2010; Ida et al., 2011; Beshel and Zhong, 2013; He et al., 2013a; Kacsoh et al., 2013; Erion et al., 2016; Kim et al., 2017; Liu et al., 2019; Tsao et al., 2018; Zhang et al., 2019). Similar to its mammalian homolog NPY, *Drosophila* NPF system regulates male sexual behavior (Liu et al., 2019; Zhang et al., 2019), ethanol consumption and sensitivity (Wen et al., 2005; Shohat-Ophir et al., 2012; Kacsoh et al., 2013), feeding behavior (Kim et al., 2017; Tsao et al., 2018), appetitive memory (Krashes et al., 2009), arousal and sleep (He et al., 2013b; Chung et al., 2017). While most studies in the field focused on NPF-producing neurons, less is known about NPF-receptor neurons and the molecular basis for their diverse functions.

In this work, we investigated the transcriptome of NPFR neurons, comparing it to those of nine other neuronal populations, and discovered that NPFR neurons have a unique signature that is enriched in neuropeptide and neuromodulator receptors. We tested the functional relevance of their transcriptome and proteome by disturbing two molecular systems that regulate large number of cellular targets: RNA editing and protein ubiquitination. Adenosine-to-inosine (A-to-I) RNA editing, is a cellular mechanism that generates transcriptomic and proteomic diversity by recoding certain adenosines within pre-mRNA sequences into inosines, leading to a variety of consequences that include amino acid sequence changes in proteins (Keegan et al., 2005; Stapleton et al., 2006; Jepson and Reenan, 2009; Rosenthal and Seeburg, 2012; Maldonado et al., 2013; Li et al., 2014). Protein ubiquitination is a highly regulated post-translational cellular mechanism that shapes protein abundance and function (Schnell and Hicke, 2003; Callis, 2014). Our results show that manipulating the transcriptome and proteome of NPFR neurons enhance certain aspects of male-female and male-male interactions, suggesting a role for NPFR neurons in restraining social and sexual behaviors.

## Results

To explore the connection between transcriptional programs in NPFR neurons and behavior, we used a recently generated dataset from our lab, that was used to profile spatial RNA editing across the fly brain (Sapiro et al., 2019). The dataset consists of RNA sequences from several neuronal populations in the brain that were obtained by immunoprecipitation of genetically tagged nuclei (INTACT method) (Sapiro et al., 2019). The dataset comprises of nine neuronal populations that are known to regulate various motivational behaviors: neuromodulatory neurons, including dopaminergic neurons (*TH-Gal4* marking 515 cells), octopaminergic neurons (the fly homolog of mammalian norepinephrine, *Tdc2-Gal4* marking 265 cells), serotonergic neurons (*TRH-Gal4* marking 989 cells), Corazonin neurons (structurally related to mammalian GnRHs, *CRZ-Gal4* marking 300 cells), NPF neurons (*NPF-Gal4* marking 41 cells), Dh44 neurons (*CRF* ortholog, *DH44-Gal4* marking 6 cells) and neurons, which express receptors for NPF (*NPFR-Gal4* marking 100 cells). Two additional population of neurons, that harbor larger number of cells were analyzed; mushroom body neurons involved in learning and memory (*OK107-Gal4* marking 2,000 cells), and fruitless-expressing neurons, that are known to regulate sex specific behavior (*Fru-Gal4* marking 1,454 cells).

Analysis of transcriptomic datasets offers a way to compare the levels of transcription per gene across different cell populations, or within the same cells under different conditions. To explore the transcriptomic landscape of NPFR cells, we took two complementary approaches: pairwise comparison of gene expression profiles between each neuronal population and all neurons (pan-neuronal driver, *Elav-Gal4*); and pairwise comparison of gene expression profiles between each neuronal population and NPFR neurons.

### The Transcriptomes of NPFR, Fru, and OK107 Neurons Are Most Similar to Those of the General Neuronal Population

**FIGURE 1:**
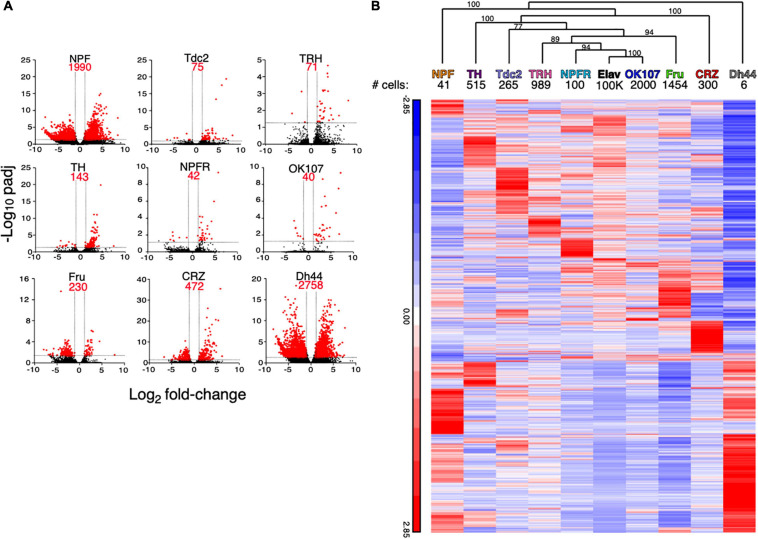
Different neuronal populations exhibit varying number of differentially expressed genes when compared to the general population of neurons. **(A)** Volcano plots of log_2_ average fold change per population of all genes (black) and significantly expressed genes (red) compared to ElaV. Dashed lines indicate thresholds for fold change and adjusted *p*-values. **(B)** Hierarchical clustering of average normalized reads for all significantly expressed genes in 9 neuronal populations compared to a pan neuronal driver (ElaV). Clustering analysis was performed using Partek. Hierarchical clustering dendrogram with *p*-values. Values on the edges of the clustering are AU (Approximately Unbiased) *p*-values. Clusters with AU ≥ 95% are considered to be strongly supported by data.

Starting with the first approach, we generated a list of differentially expressed genes (DEGs) for each neuronal population with significantly different expressions than those in all neurons (greater than twofold change compared to the expression in ElaV and have an adjusted *p*-value smaller than 0.05) (Figures 1A,B and Supplementary Table 1). Since the number of DEGs in each neuronal population represents the difference in transcriptome between this population and all neurons, we expected that the more specific the transcriptome in a population is, the more unique it will be compared to ElaV. Interestingly, DH44- and NPF-expressing neurons displayed the largest number of DEGs (2,758 and 1,990, respectively), while OK107- and NPFR-expressing neurons presented the smallest number of DEGs (40 and 42, respectively) (Figure 1A). Most DEGs in OK107, NPFR, TRH, Tdc2, and TH were found to be over-expressed compared to those in ElaV, while most DEGs in Fru neurons were under-expressed compared to those in ElaV (Figure 1A). Hierarchical clustering analysis of average normalized reads for all the DEGs between the different neuronal populations (union of all cell type specific DEGs) confirmed this finding: DH44 cells were clustered apart from all other populations, followed by NPF cells (Figure 1B); in addition, OK107 cells clustered closest to ElaV, and NPFR neurons are located next to the OK107-ElaV cluster (Figure 1B). Altogether, this suggests that the transcriptomes of DH44- and NPF-expressing cells are the most unique, whereas those of OK107-, and NPFR-expressing neurons resemble the general neuronal population.

### Shared DEGs Between Neuronal Populations Reveal a Complex Pattern

**FIGURE 2:**
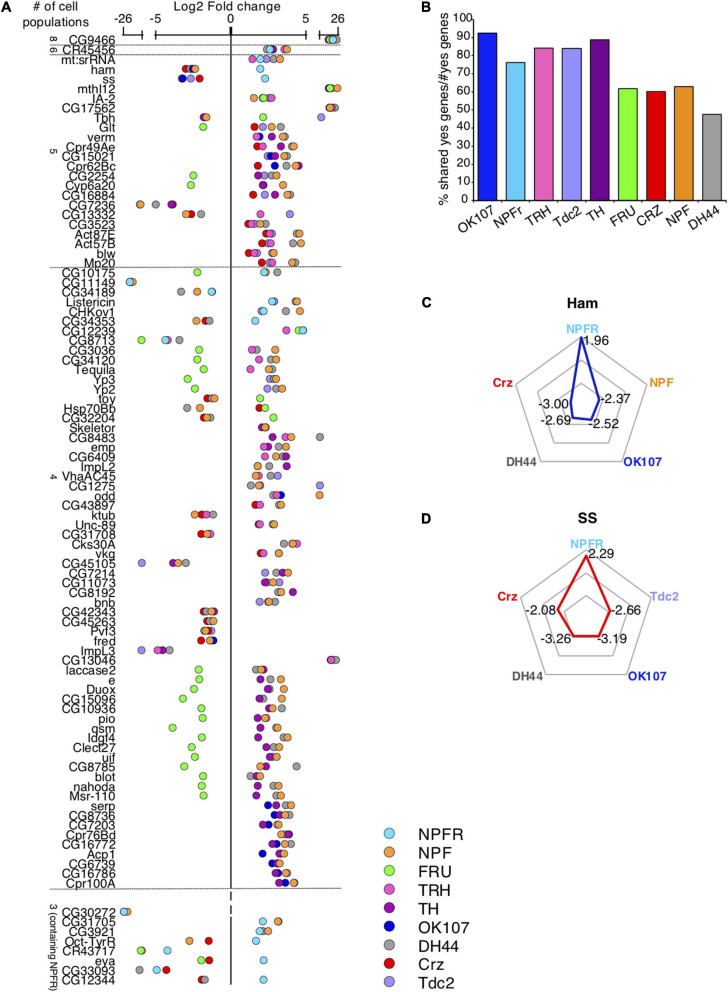
Certain DEGs are shared across many populations. **(A)** Scatter plot representing log_2_-fold change of all genes that are differentially expressed compared to ElaV and are shared across 8–4 different cell populations (upper part) and across 3 populations containing NPFR (bottom part). **(B)** Percent of shared DEGs normalized by total number of DEGs in each neuronal population. **(C,D)** Radar plots of two DEGs: ham **(C)** and ss **(D)** both of which were enriched in NPFR cells compared to 4 other cell types.

Given the partial anatomical overlap between several neuronal populations in our dataset (Certel et al., 2010; Andrews et al., 2014; Shao et al., 2017; Croset et al., 2018; Davie et al., 2018; Liu et al., 2019), we next asked whether some DEGs are shared across different neuronal populations. Enrichment or depletion of the same genes in more than one population suggests that these neuronal populations share differences from the general population, and/or that some of their neurons overlap. Searching for DEGs that are shared by different neuronal populations, we did not document any genes that are shared by all nine populations (Figure 2A, Table 1 and Supplementary Table 2). When comparing shared DEGs across 8-3 neuronal populations, only a single gene (CG9466) was found to be shared by eight populations, exhibiting similar pattern of enrichment in all eight populations (Figure 2A and Supplementary Table 2). The long non-coding RNA CR45456 is another example for a transcript that is enriched in six neuronal populations when compared to its expression in ElaV (Figure 2A and Supplementary Table 2).

**TABLE 1:** The number of shared DEGs varies across populations.

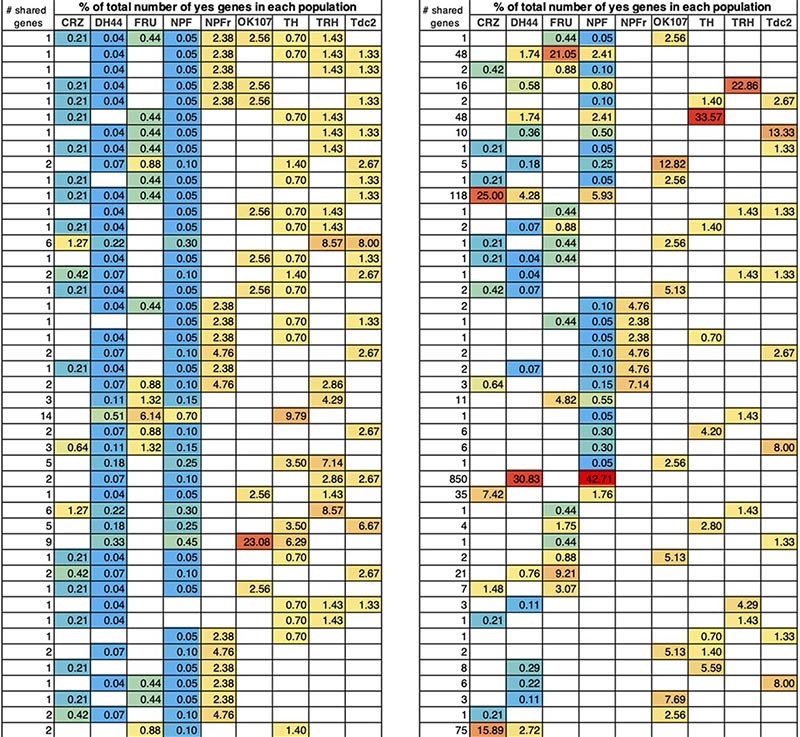

**Number of shared DEGs across populations (left column) represented as % from the total number of DEGsin each population (color coded: red-high, blue-low).**

Two neuronal populations shared the largest number of DEGs with all other populations: NPF and DH44 (1,253 genes in 67 comparisons and 1,309 genes in 56 comparisons, respectively, Figure 2A, Table 1, and Supplementary Table 2). The number of DEGs varied across all populations by two orders of magnitude (Figure 1A), increasing the odds for shared DEGs in certain populations due to the overall number of DEGs and not because they were expressed within overlapping neurons. To control for this, we normalized the number of shared DEGs by the total number of DEGs in each population and found a reduction in the variation of the numbers of shared DEGs between populations (Figure 2B). This finding implies that the probability of sharing a DEGs is similar across different populations, and that the more DEGs a population has, the higher the probability that some will be shared, emphasizing the need to use other criteria to determine whether two populations share similar transcriptional patterns or just mutual neurons.

Interestingly, and although Fru shares neurons with several other populations, such as NPF and Tdc2 (Certel et al., 2010; Andrews et al., 2014; Liu et al., 2019), as evidenced by the enrichment of Tbh (Tyramine β hydroxylase) in Fru and Tdc2 neurons, most DEGs in Fru neurons were depleted compared to their expressions in other populations (Figure 2A). Striking examples are Cyp6a20, Glutactin, Tequila, and quasimodo, which support the notion that most Fru neurons are distinct from the rest of the analyzed neuronal populations. In addition, CRZ-, DH44- and NPF-expressing neurons shared similar expression patterns of groups of genes that shape neurophysiology, possibly due to all of them being peptidergic neurons. Examples of these neurophysiology-associated genes include: the shared patterns of ion channels, such as NaCP6OE (Voltage gated Na channel), Teh1 (TipE homolog 1 sodium transport regulation), genes involved in neuronal signaling, such as Neuroligin 3 (synaptic adhesion molecule), beat-1C (beaten path 1C axon guidance), Tehao (Toll signaling); and the shared patterns of receptors, such as nicotinic acetylcholine receptor alpha3 and 6, Toll6 (Toll-like receptor family), IR47a + b (ionotropic receptor a + b), GluR1A (Glutamate receptor 1A), and Oct-beta-3R (Octopamine receptor beta 3).

The shared DEGs between NPFR neurons and other neuronal populations illuminated a complex pattern of 21 genes that are similarly and oppositely expressed (Figure 2A). The two most differentially regulated genes were hamlet (ham) and spineless (ss), both highly enriched in NPFR neurons and depleted in all other neuronal populations (Figures 2C,D). Examining shared DEGs in comparison to NPF neurons revealed two more genes with opposite expression that are enriched in NPFR (Octopamine-Tyramine Receptor and CG34353) and 11 DEGs with similar expression. NPFR neurons also displayed expression patterns of DEGs different from those in DH44 neurons, with four oppositely expressed DEGs, including ham, ss, CG34353 and CG12344, and similarly expressed genes, like CG9466, CR45456, mt:srRNA, CG10175, CG34189, Listericin, CHKOV1, CG12239, CG8713, CG31705, CG3921, CR43717, and CG33093 (Figure 2A). Interestingly, Octopamine-Tyramine Receptor (Oct-TyrR), which is regulated by feeding and mediates appetitive changes in locomotion (Schützler et al., 2019), was enriched in NPFR-expressing neurons and depleted in NPF- and CRZ-expressing neurons (Figure 2A). Furthermore, ss, which encodes a transcription factor regulating female receptivity to male courtship (Mcrobert, 1991), was enriched in NPFR neurons. This data suggests that while it is possible that some of the NPF and DH44 neurons share neuronal subpopulations with NPFR, many of the neurons in these populations do not overlap.

**FIGURE 3:**
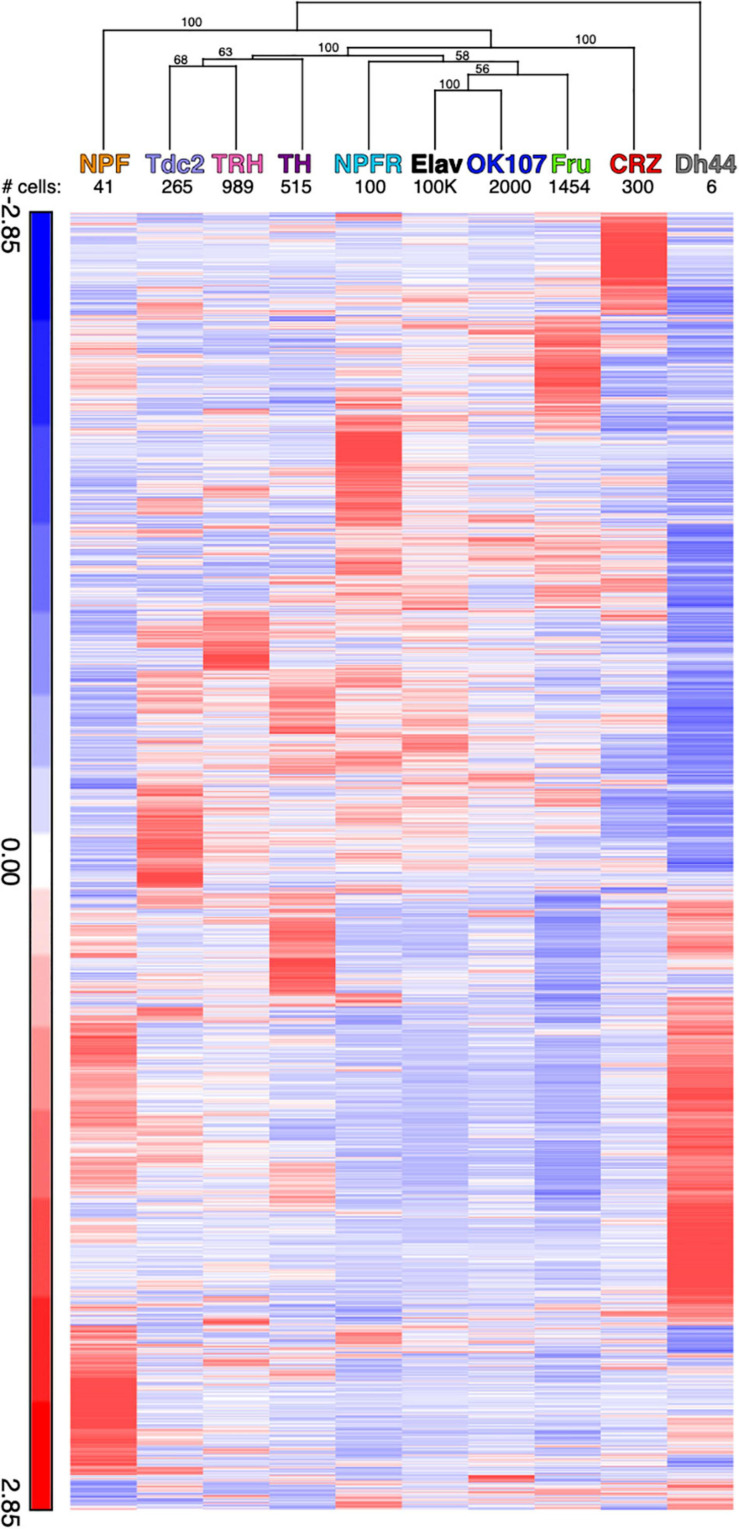
Hierarchical clustering of average reads for all differentially expressed genes compared to the NPFR neurons. Clustering analysis was performed using Partek. Hierarchical clustering dendrogram with *p*-values. Values on the edges of the clustering are AU (Approximately Unbiased) *p*-values. Clusters with AU ≥ 95% are considered to be strongly supported by data.

Next, we analyzed the relative expression patterns of NPFR neurons using the second pairwise approach, comparing NPFR neurons to each of the neuronal populations (Figure 3 and Supplementary Table 3). The pairwise comparison of NPFR to ElaV expression profiles resulted in the identification of 42 DEGs, but comparing the expression pattern of NPFR neurons to those of all other populations revealed a larger number of differentially expressed genes than when compared to ElaV neurons, with the exception of DH44 neurons. Hierarchical clustering of the identified DEGs in each of the comparisons, followed by boot strapping analysis revealed that DH44- and NPF-expressing neurons clustered away from the rest of the populations, while Fru and OK107 neurons were most similar to NPFR neurons (Figure 3). In addition, Crz, NPF, and DH44 neurons clustered further from NPFR, while TH, TRH, Tdc2 clustered apart from Ok107, Fru, Elav, NPFR.

**FIGURE 4:**
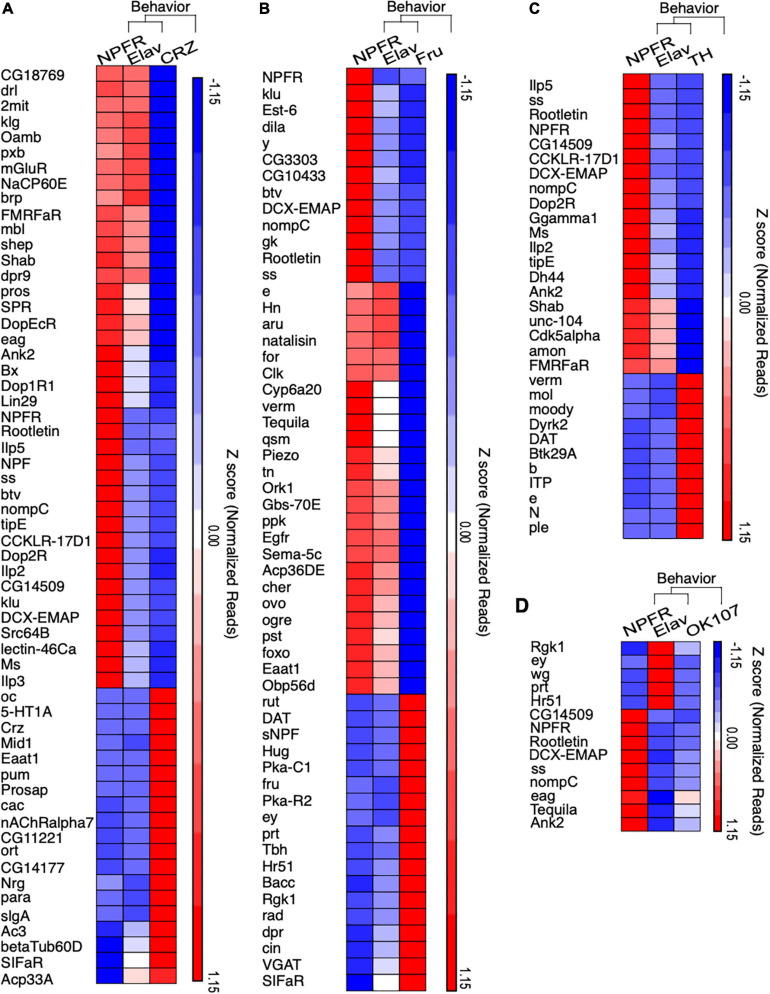
NPFR expressing neurons exhibit enrichment of behavior related genes. **(A–D)** Hierarchical clustering of statistically overrepresented biological processes that are related to behavior in differentially expressed genes of NPFR expressing neurons compared to Corazonin (CRZ, **A**), Fruitless (FRU, **B**), Dopamine producing (TH, **C**) neurons and Mushroom bodies neurons (OK107, **D**). Biological overrepresentation was performed using PANTHER. Clustering analysis was performed using Partek.

To further explore the biological relevance of the identified DEGs, we used a statistical overrepresentation analysis (PANTHER), which highlighted several biological processes, including enrichment of genes associated with regulation of behavior (Figure 4 and Supplementary Table 4). We focused on behavior-associated genes that were enriched or depleted in NPFR vs. CRZ, TH, Fru, and OK107 neurons and found some interesting patterns (Figure 4). NPFR neurons displayed enrichment of genes that mediate different forms of learning and memory, such as derailed, 2mit, klingon, CG18769, Oamb, mGluR (metabotropic Glutamate Receptor), eag, Ank2, ss, and Tequila (Figure 4). In addition, we identified enrichment of genes involved in sensory perception of sound and touch, such as Ank2, btv, nompC, CG14509, DCX-EMAP, dila, and Rootletin. Interestingly, we documented enrichment of a few genes that participate in insulin signaling, such as dilps 2, 3, and 5 in Dh44 neurons, suggesting an anatomical overlap between some NPFR neurons and insulin-producing cells (IPCs).

Intriguingly, NPFR neurons exhibited enriched levels of various receptors for neuropeptides and neuromodulators like Oamb, mGluR, Dop1R1, Dop2R, CCKLR-17D1, Lestin-46Ca, Ms, TrissinR, CCHa1-R (CCHamide-1 receptor), AstA-R1(Allatostatin A receptor1), rk (rickets), Proc-R (Proctolin receptor), SPR (sex peptide receptor), sNPF-R, and (of course) the receptor for NPF (Figure 4 and Supplementary Figure 2). The enrichment of such diverse types of receptors indicates that NPFR neurons receive multiple inputs from many neuromodulator systems, and/or that they are composed of diverse groups of neurons, with distinct combinations of receptors. In any event, these findings support the hypothesis that NPFR neurons are located at a convergence point of information that is relevant for the integration of internal state and action selection.

Lastly, we found that NPFR neurons possessed a unique mixture of ion channels compared to both Crz and Fru neurons (Supplementary Figure 3), with an overrepresentation of seven potassium and sodium ion transmembrane transport subgroups in NPFR neurons (Supplementary Table 4). We also documented an overrepresentation of amino acid transmembrane transport proteins with 2 enriched genes in Fru cells (vGAt, CG5549) and 6 enriched in NPFR neurons (Ncc69, CG7888, Eaat1, CG43693, CG8785, CG16700). Interestingly, Orct2 (Organic cation transporter 2), which is a transcriptional target of the insulin receptor pathway (Herranz et al., 2006; Supplementary Figure 3) was enriched in NPFR compared to Crz, further supporting the involvement of NPFR neurons in insulin signaling.

### Manipulation of the Proteome Profile in NPFR Neurons Affects Social and Sexual Behavior in Flies

The distinct patterns of transcription in each neuronal population gives rise to a specific proteome diversity that shapes the functional output of neurons. To investigate this assumption further, one can modify the expression levels of genes that are enriched or depleted in certain populations or use a more global approach to disturb the proteomic signature of specific neurons. We chose to perturb the transcriptomic and proteomic signature of NPFR neurons by manipulating the function of two molecular systems that regulate a large number of cellular targets (RNA editing and protein ubiquitination) and to analyze the effects on the social behavior of male flies.

**FIGURE 5:**
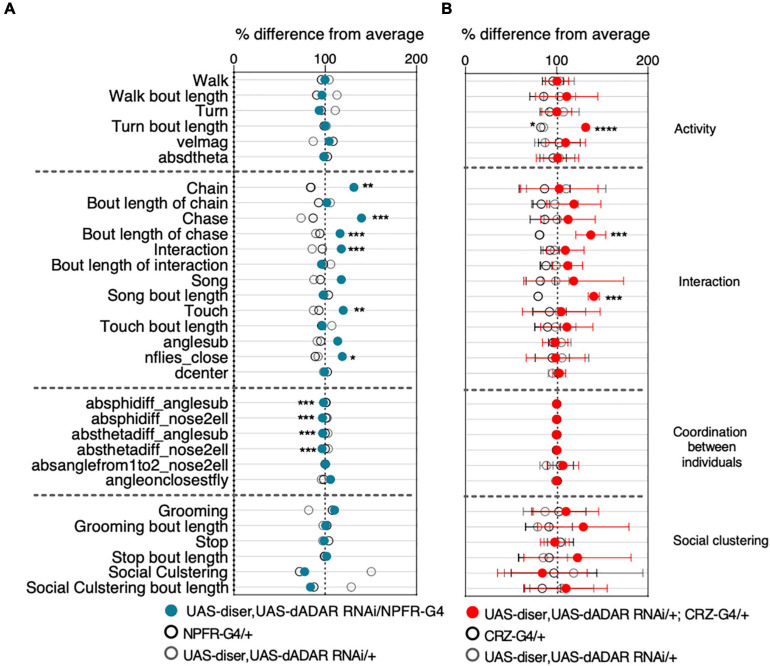
RNA editing is required in NPFR neurons for typical male social behavior. Behavioral signatures of social group interaction. Data is presented as normalized scatter plots depicting% difference from average of 31 behavioral features. **(A)** Behavior profile of male flies harboring NPFR-Gal4/UAS-Dicer, UAS-dADAR RNAi (Blue) compared to genetic controls (UAS-Dicer, UAS-dADAR RNAi/ +, and NPFR-Gal4/ +, gray and black, respectively). *n* = 17. **(B)** Behavior profile of male flies harboring UAS-Dicer, UAS-dADAR RNAi/ +; CRZ-Gal4/ + (red) compared to genetic controls (UAS-Dicer, UAS-dADAR RNAi/ +, CRZ-Gal4/ +, gray and black, respectively). *n* = 7. One-way ANOVA with Tukey’s *post hoc* for normally distributed parameters and Kruskal-Wallis with Dunn’s test *post hoc* for non-normally distributed parameters. FDR correction was performed for all features. *P < 0.05, **P < 0.01, ***P < 0.001, ****P < 0.0001, ^n.s.^*P* > 0.05.

Thousands of RNA editing sites have been discovered in *Drosophila* (Rosenthal and Seeburg, 2012), most of which lead to recoding events in genes that are expressed and function specifically in the neuron (Keegan et al., 2005; Stapleton et al., 2006; Jepson and Reenan, 2009; Rosenthal and Seeburg, 2012; Maldonado et al., 2013; Li et al., 2014). As such, null mutation of ADAR in *Drosophila* leads to strong locomotor phenotypes that become more severe with age, although the underlying mechanisms is still mostly unknown (Palladino et al., 2000). Therefore, we hypothesized that reducing ADAR expression in NPFR neurons would affect the proteomic profile and could result in behavioral phenotypes. To test this, we downregulated the expression of dADAR in NPFR neurons (NPFR > UAS-dicer, UAS-dADAR RNAi) and analyzed behavior in groups of 10 flies, using the “FlyBowl” system, a suite of tracking and behavior analysis software that score plethora of locomotion and social behaviors (Robie et al., 2012; Bentzur et al., 2021). We used the tracking data obtained to generate a comprehensive behavioral representation for experimental flies and genetic controls that included kinetic features and eight complex behaviors. The overall differences between the genotypes are depicted in a scatter plot of normalized differences, divided into four main categories: activity-related features, interaction-related features, coordination between individuals, and social clustering-related features (Figure 5A and Supplementary Figure 1).

Unlike dADAR null flies and pan-neuronal knockdown (KD) of dADAR flies, which display strong motor impairments, downregulation of dADAR expression in NPFR neurons did not lead to any differences in locomotion and general activity levels. Specifically, the average velocity of experimental flies and the percentage of time they spent walking and performing body turns was similar to those of the genetic controls (NPFR-Gal4/+ and UAS-dicer, UAS-dADAR RNAi/+, Figure 5A). We further analyzed several types of social behaviors, including touch (active leg touching between two flies), approach (fly approaching another fly), song (wing extension and vibration to generate male courtship song), chase (fly chasing another fly), and chaining (one fly following a fly while being followed by another fly, in a minimum chain length of three flies). Interestingly, reducing ADAR levels in NPFR neurons resulted in strong elevation in social interaction between male flies, as manifested by increased levels of close touch behavior, increased levels of song display, increased values of active approaches and male-male chase events that resulted in multiple formations of chains (Figure 5A). In addition to these behaviors, we analyzed another feature associated with social interaction: the number of flies close-by (nflies-close), representing the number of flies within two body lengths of a focal fly as a measure of sociality (Figure 5A and Supplementary Figure 1). Flies harboring reduced levels of ADAR in NPFR neurons depict significantly higher value of nflies-close compared to the control groups, suggestive of close distance between flies (Figure 5A), altogether indicating that RNA editing in NPFR expressing cells is important for the correct expression of certain social behaviors. A previous study in our lab demonstrated that NPFR and CRZ neurons possess distinct RNA editing profiles (Sapiro et al., 2019). This prompted us to test the behavioral significance of reducing RNA editing in CRZ neurons as well. However, knocking down ADAR in CRZ neurons only led to moderate effects on the male-male social interactions. Specifically, we documented longer bouts of song, turn, and chase events than in genetic controls (Figure 5B).

**FIGURE 6:**
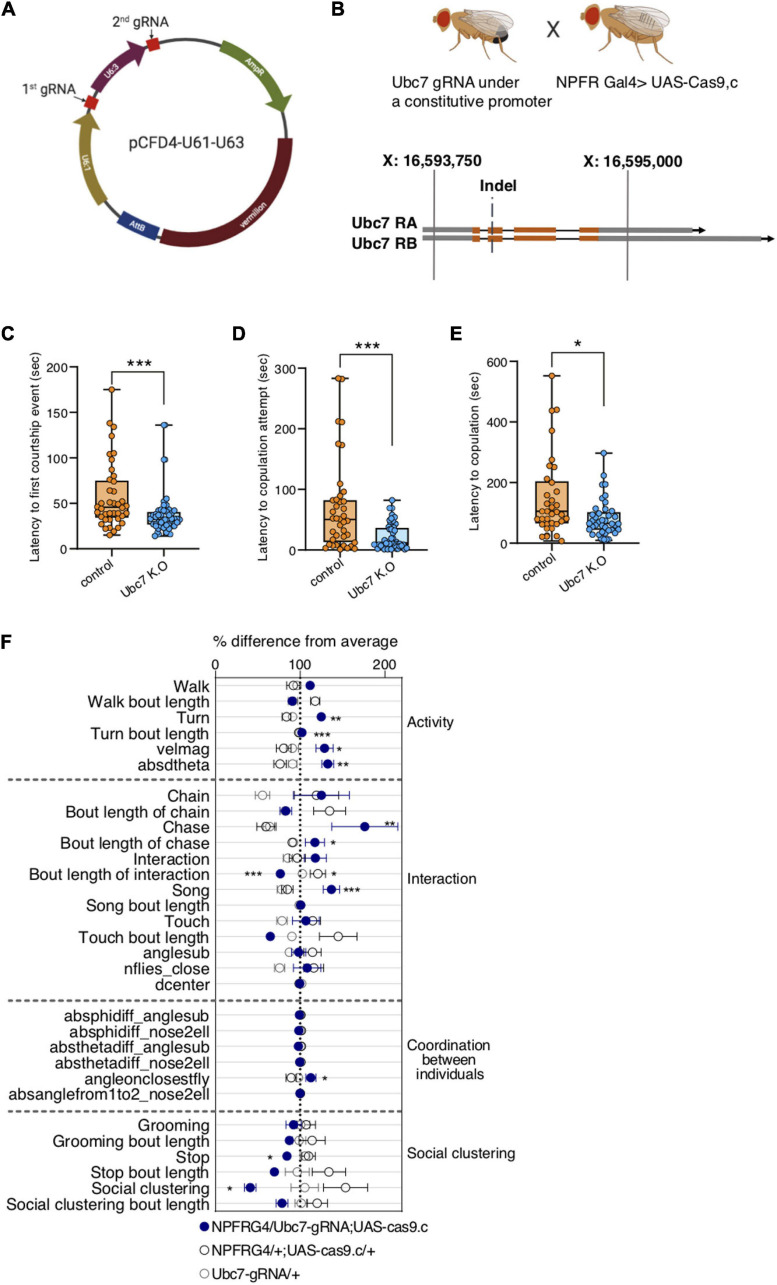
Tissue specific K.O of Ubc7 elevates the motivation to court and enhances male-male social interaction. **(A)** Representation of pCDF4 plasmid containing two gRNAs (red). **(B)** Crossing scheme of flies containing gRNAs with NPFR-Gal4; Cas9.c flies to generate tissue specific Ubc7 K.O flies (upper panel). Lower panel depicts two Ubc7 isoforms (orange and gray blocks representing coding and non-coding exons, respectively, Black lines representing introns). The double strand break occurred at the beginning of the 2nd coding exon. **(C–E)** Male flies containing NPFR-Gal4/Ubc7-gRNA; UAS-Cas9.c/ + (blue) were introduced to naïve females in courtship arenas and were video recorded, their courtship behavior was analyzed for latency to first courtship event **(C)**, latency to first copulation attempt **(D)** and latency to copulation **(E)** compared to genetic controls (NPFRG4/attp1;UAScas9.c, orange). *n* = 47 and 40 in **(C)**, *n* = 46 and 39 in **(D)**, *n* = 39 and 33 in **(E)** for experimental and control groups, respectively. Mann-Whitney test. *P < 0.05, ***P < 0.001. **(F)** Behavioral signatures of social group interaction in male flies harboring NPFR-Gal4/Ubc7-gRNA; UAS-Cas9.c/+ (blue) compared to NPFR-Gal4/+; UAS-Cas9.c/+ and Ubc7 gRNA/+ (gray and black, respectively). Data is presented as normalized scatter plots depicting % difference from average of 31 behavioral features. *n* = 8, 5, and 11, respectively. One-way ANOVA with Tukey’s *post hoc* for normally distributed parameters and Kruskal-Wallis with Dunn’s test *post hoc* for non-normally distributed parameters. FDR correction was performed for all features. *P < 0.05, **P < 0.01, ***P < 0.0001, ^n.s.^*P* > 0.05.

Next, we perturbed the proteome diversity in NPFR cells by targeting the protein ubiquitination machinery. To manipulate this multiplayer system, we targeted the expression of one central player, ubiquitin-conjugating enzyme 7 (Ubc7), orthologous to the human ubiquitin-conjugating enzyme E2 G2. We used the CrispR-Cas9 system to generate tissue specific knockout (KO) of Ubc7 using a combination of Ubc7-specific guide RNAs and specific expression of Cas9 in NPFR neurons (NPFR > UAS Cas9) (Xue et al., 2014; Meltzer et al., 2019; Poe et al., 2019). We generated a pair of guide-RNAs (gRNA) targeting the beginning of the second exon of Ubc7. We validated their efficiency by using a germline deletion within the Ubc7 locus by driving Cas9 expression using a germline-specific driver (Vas-Cas9), which resulted in an 18- to 23-bp deletion at the beginning of the second exon of both Ubc7 isoforms (Figure 6A and Supplementary Figure 4). To affect Ubc7 in NPFR cells, we crossed *NPFR*-G4; *UAS-Cas9*.c flies with flies carrying our gRNA for Ubc7. Since Ubc7 null mutation was shown to suppress courtship toward females (Orgad et al., 2000), we first analyzed the effects of knocking out Ubc7 in NPFR neurons on male courtship behavior. For that, we introduced experimental male flies (NPFR > UAS Cas9, *gRNAs*) or genetic control male flies (*NPFR-G4/attp1; UAS-Cas9.c*) into courtship arenas with virgin females and recorded and analyzed their behavior (Figures 6C–E). Surprisingly, and in contrast with Ubc-7 null mutants, male flies lacking Ubc7 expression in NPFR cells displayed shorter latency to court, shorter latency to first copulation attempt, and shorter time to successful copulation (Figures 6C–E), all signs suggestive of higher motivation to court and mate. Next, we analyzed the behavioral responses of male flies when interacting with nine other male flies in a group (Figure 6F). Contrary to the previous results obtained after manipulating the proteome of NPFR by disturbing RNA editing programs, which did not affect any of the measured activity-related features, knocking out Ubc7 in NPFR neurons led to a pronounced increase in the number of time flies spent walking and performing turns, and to an overall increase in their average velocity compared to genetic controls (Figure 6F), suggestive of increased arousal. Moreover, Ubc7 KO male flies exhibited increased social interactions between males, as shown by the higher levels of chase and song and reduced social clustering (Figure 6F). This suggests, that protein ubiquitination in NPFR neurons is important for regulating the intensity of male-female and male-male sexual and social behaviors, and that Ubc7 is necessary to reduce male social interactions.

## Discussion

The complex interplay between genes, neurons and behavior started to be deciphered decades ago with the Benzerian revolution in neurogenetics and is still under intense investigation these days using a plethora of tools in various model organisms. This study joins a growing body of studies that use contemporary genomic approaches to dissect the brain into units and illuminate their molecular content, as a step toward understanding the dynamic spatiotemporal environments in which genes function (Croset et al., 2018; Agrawal et al., 2019; Shih et al., 2019). While many cell types exist in the fly brain, in this study, we analyzed only a small fraction of them, focusing mostly on NPFR neurons. Nevertheless, the transcriptome profiles of other neuronal populations in this dataset can serve as a resource for labs investigating other neurons.

We took two complementary pairwise based approaches to investigate the relative signature of NPFR neurons: the first approach comparing profiles of each of the populations to those of all neurons and subsequently comparing the DEGs across populations; and the second approach performing pairwise comparisons between NPFR and each of the nine neuronal populations. Although the two approaches highlighted different number of differentially expressed genes, they resulted in similar hierarchical clustering patterns, and complemented the picture describing the distinct molecular landscape of NPFR neurons.

By comparing expression profiles of different neuronal populations to those of all neurons, we found that NPF expressing neurons represent a much more unique population than NPFR neurons. This may result from differences in cell number (40 NPF vs. ∼100 NPFR cells) or may be associated with the heterogenous expression profile of NPFR as receptor neurons. The second explanation is supported by the enriched levels of receptors for neuropeptides and neuromodulators we detected, a finding that is in agreement with those of previous studies showing anatomical overlap between NPFR cells and some NPF and TH neurons (Shao et al., 2017; Zhang et al., 2019). Still, it is not known if NPFR neurons receive multiple inputs from many neuromodulatory systems, or whether they are composed of diverse groups of neurons with distinct combinations of receptors. This question could be addressed in future studies by dissecting NPFR neuronal population into smaller subsets of cells using genetic intersection approaches or by single cell RNA-seq analysis of NPFR positive neurons.

The second part of this study investigated the functional relevance of the unique transcriptome identified with our genomic approach. We discovered that global perturbation of RNA editing and protein ubiquitination programs in NPFR neurons resulted in dramatic behavioral phenotypes. Tissue-specific knockout of Ubc7 in male flies resulted in a strong motivation to court female flies that could possibly stem from increased level of arousal as reflected by enhanced velocity. The enhanced courtship display found upon the tissue specific knockout of Ubc7 is in contrast with the complete loss of courtship behavior documented in male flies that lack Ubc7 in all cells (Orgad et al., 2000). This apparent discrepancy can be easily explained by distinct roles of Ubc7 in different tissues, the lack of which in NPFR possibly perturb the proper function of NPFR in restraining courtship as shown by Liu et al. (2019). Interestingly, a subset of NPFR-dopamine neurons has been shown to promote mating drive (Zhang et al., 2019), strengthening the notion that different sub-populations of NPFR neurons have distinct roles in regulating the motivation to court, and stressing the need for dissecting NPFR cells into smaller groups of neurons to analyze their transcriptomes and functions.

In addition to the increased motivation to court, we also documented increased frequencies of male-male social interactions, including increased levels of song and chase behaviors, which are normally absent in socially experienced male flies that are housed in groups (Bentzur et al., 2021). This manifestation, together with the increased walking velocity and lack of social clustering behavior observed in groups of Ubc7 KO flies, resemble the behavioral properties of male flies exposed to social isolation, a condition that is known to promote aggression (Wang et al., 2008; Liu et al., 2011). Given that the physical features of the FlyBowl set-up prevent the expression of aggression displays such as lunging, the increased chase behavior documented in the FlyBowl setup may be indicative of the male-male aggressive behavior that is normally suppressed by NPF action on NPFR neurons (Dierick and Greenspan, 2007) and that was promoted by perturbing the proteome balance in NPFR neurons.

We have previously shown that different neuronal populations possess unique RNA editing profiles (Sapiro et al., 2019), suggesting that RNA editing may account for some functional differences between neuronal populations in the brain. The pronounced behavioral outcome of perturbing RNA editing in NPFR neurons supports this hypothesis and shows that RNA editing is necessary for the proper function of these neurons. The phenotypic resemblance to Ubc7 K.O in NPFR neurons suggests that both manipulations perturb the function of NPFR in regulating social interaction, but closer inspection reveals the existence of interesting differences. While perturbing protein ubiquitination affects activity/arousal that may stimulate chase behavior, lack of RNA editing leads to pronounced increase in approach behavior, interaction, chase and chaining behaviors without changing activity levels. These differences suggest that perturbing RNA-editing and protein ubiquitination do not lead to global malfunction of NPFR but rather affect distinct targets that regulate different features of NPFR physiology and function.

The behavioral phenotypes of reducing ADAR levels were more pronounced in NPFR than in CRZ neurons, suggesting that RNA editing does not have a uniform role in all neurons but rather shapes the diversity of expressed proteins in different neurons to allow their distinct function. Our findings join a previous study that demonstrated the spatial requirements of ADAR expression in regulating locomotor behavior (Jepson and Reenan, 2009), emphasizing the need to extend this to other behavioral paradigms, neuronal populations and even to studying the tissue specific role of specific editing events.

To conclude, in this study we demonstrated that the function of NPFR neurons in suppressing certain aspects of social behavior depends strongly on the integrity of its transcriptome and proteome. This finding highlights the importance of cell specific posttranscriptional mechanisms in regulating the abundance and function of certain RNA molecules and proteins, the action of which determines the output function of the neuron. Based on the function of the NPF/R system in regulating other types of motivational behavior we expect the unique transcriptome of NPFR to regulate also feeding behaviors, appetitive memory, ethanol related behaviors and sleep.

Considering the technology driven revolution in deciphering the connectome of the fly brain (Zheng et al., 2018; Scheffer et al., 2020), the next challenge in understanding the neurobiology of complex behavior will be to combine these static 3D maps with the molecular programs that function within defined circuits. This will be especially important for understanding a long-standing question of how a given circuit is shaped by context and internal states to produce different outcomes from a seemingly similar input? A good starting point toward solving this question will be to focus on circuits that are regulated by neuromodulators, such as NPFR neurons, and use cell specific transcriptomics to identify dynamic changes in expression pattern under various conditions corresponding to different internal states. Newly emerging technologies that allow the profiling of smaller amounts of cells (Croset et al., 2018) and spatially resolved transcriptomics (Alon et al., 2021) can open the way for identifying key cellular pathways that encode changes in motivation. Studying the functional relevance of such regulatory events requires manipulating their expression in the most accurate spatial-temporal context as best demonstrated by the different outcomes of eliminating Ubc7 expression in the whole animal vs. specifically in NPFR neurons.

Lastly, the similar patterns of social responses observed across the animal kingdom suggest, that certain social behaviors originated early in evolution, and that similar ancient biological principles and genes are involved in these processes. An example for this is the functional conservation of the NPF/R system from worms to humans in regulating social behavior, feeding, sleep-wake, ethanol related behaviors and the response to stress (De Bono and Bargmann, 1998; Tokuno et al., 2002; Rogers et al., 2003; Sokolowski, 2003; Davies et al., 2004; Heilig, 2004; Kalra and Kalra, 2004; Karl and Herzog, 2007; Sparta et al., 2007; Briggs et al., 2010; Xu et al., 2010; Wiater et al., 2011; Shohat-Ophir et al., 2012; Chung et al., 2017; Robinson and Thiele, 2017; Li et al., 2018). While the transcriptome of NPF and NPFR neurons support this notion and illuminate some of the cellular pathways participating in these behaviors (Ubc7 for courtship and the insulin pathway for the regulation of feeding), further work is needed to comprehensively decipher their function and extend these findings beyond flies.

## Materials and Methods

### Fly Lines and Culture

*Drosophila melanogaster* CS flies were kept in 25°C, ∼50% humidity, light/dark of 12:12 h, and maintained on cornmeal, yeast, Molasses, and agar medium. NPFR-GAL4 was a gift from the Truman lab (HHMI Janelia Campus), CRZ-GAL4 was a gift from the Heberlein lab (HHMI Janelia Campus), UAS-dicer, UAS-dADAR RNAi was a gift from the Lee lab (Stanford University), UAS-CAS9.c was a gift from the Schuldiner lab (Weizmann Institute). Vasa-CAS9 was a gift from Gershon lab (Bar-Ilan University), y^1^w^67c23^;P{CaryP}attP1 (BestGene BL#8621).

### Determining Gene Expression Levels From RNA-Seq

Previously published RNA-seq data was used (Sapiro et al., 2019). Reads were trimmed using cutadapt (Martin, 2011) and mapped to *Drosophila melanogaster* (BDGP6) genome using STAR (Dobin et al., 2013) v2.4.2a (with EndToEnd option and out FilterMismatchNoverLmax was set to 0.04). Counting proceeded over genes annotated in Ensembl release 31, using htseq-count (Anders et al., 2015) (intersection-strict mode). DESeq2 (Love et al., 2014) was used to measure differential expression analysis with the betaPrior, cooks Cutoff and independent Filtering parameters set to False. Genes were filtered in a pairwise manner according to the following parameters: log_2_-fold change of at least | 1|, adjusted *P*-value lowers than 0.05 (Benjamini and Hochberg procedure) and a minimum of least of 30 normalized counts in one of the repeats.

### Accession Numbers

Raw data is available at GEO with accession GSE113663.

### FlyBowl

FlyBowl experiments were conducted as described in Bentzur et al. (2021). In brief: groups of 10 male flies, which were socially raised in groups of 10 for 3–4 days, were placed in FlyBowl arenas, and their behavior was recorded at 30 fps for 15 min and were tracked using Ctrax (Branson et al., 2009). Automatic behavior classifiers and Per-frame features were computed by JABBA (Kabra et al., 2013) tracking system. Data of all behavioral features was normalized to % difference from the average of each experiment for visualization. Details about the different features are found in Supplementary Figure 1.

### Courtship Assay (ubc7)

Four to Five days old naive males were placed with 4–5 days old virgin females in round courtship arenas (0.04 cm^3^ in volume), one male and one female in each arena. Courtship arenas were placed in behavior chambers, under controlled temperature and humidity (25°C, 70% humidity). Behavior was recorded for 10 min from the introduction of male and female pairs using Point-Grey Flea3 cameras (1,080 × 720 pixels at 60 fps). Latency to copulation attempt and latency to copulation were quantified for each pair relative to the first wing vibration the male exhibited. Statistics: Mann-Whitney test.

### Generation of gRNA, Transgenic Constructs and Transgenic Flies

gRNA sequences were selected using the Fly- CRISPR algorithm^1^, contain 20 nucleotides each (PAM excluded), and are predicted to have zero off-targets. Two different gRNA sequences were selected for Ubc7, both within the coding region of the gene, but not overlapping each other. Both gRNA sequences were cloned into the pCFD4 plasmid (Figure 6A). Cloning into pCFD4 was done using Q5^®^ High-Fidelity DNA Polymerase (BioLabs). gRNA-harboring constructs were injected to Drosophila embryos and integrated into attP landing sites using the φC31 system into attP1 (BL#8621) on the second chromosome. Injections were performed as services by BestGene^2^.

gRNA sequences:
GTTAACACTTGACCCGCCCG
GCCCCATCAGCGAGGACAAC.

### Generation of the Germline ubc7 Indel Mutant

Transgenic flies expressing gRNA pCFD4 were crossed to flies expressing Vas-Cas9. Flies containing both the gRNAs and nos-Cas9 were crossed to a Fm7a balancer line, offspring were then collected and checked for the presence of an indel using DNA seq. The resulting indel is a deletion of 18–23 bp (Figure 6B and Supplementary Figure 4).

Primers for DNA sequencing:
Forward: AGAAAGCCACTCGATTCATTCGATA
Reverse: GTCCAGAGCGTGGAGAAGAT.

### Generation of Tissue Specific CRISPR

Transgenic flies expressing gRNA pCFD4 were crossed to flies expressing NPFRG4/+; UAS-Cas9.c/+.

### Statistical Analysis

Data of each behavioral feature per experiment was tested for normality and consequently tested by either One-way ANOVA or Kruskal-Wallis tests followed by Turkey’s or Dunn’s *post hoc* tests using Prism. FDR correction for multiple comparisons was performed for all features. Statistical overrepresentation was generated using PANTHER (Thomas et al., 2006; Mi et al., 2019)^3^.

Hierarchical clustering dendrogram with *p*-values was done using the R package pvclust^4^, with multiscale bootstrap resampling of 10,000 iterations to assess statistical significance, represented by a 1–100 score. Hierarchical clustering was performed using average linkage method with Euclidian distance as the distance measure.

### Graphics

Figures 6A,B were Created in BioRender.com.

## Data Availability Statement

The raw data supporting the conclusions of this article will be made available by the authors, without undue reservation.

## Author Contributions

AB, JR, AS, and GS-O: conceptualization. AB and JR: methodology and statistical analysis. JR, AB, OS, SD, MT, and ML: investigation. JR, AB, and GS-O: writing. GS-O: funding acquisition and supervision. All authors contributed to the article and approved the submitted version.

## Conflict of Interest

The authors declare that the research was conducted in the absence of any commercial or financial relationships that could be construed as a potential conflict of interest.

## Footnotes

1 http://flycrispr.molbio.wisc.edu/

2 https://www.thebestgene.com/

3 http://pantherdb.org/citePanther.jsp

4 https://academic.oup.com/bioinformatics/article/22/12/1540/207339

## Supplementary Material

Supplementary Figure 1 List of behavioral features presented in Figures 5, 6.

Supplementary Figure 2 NPFR expressing neurons exhibit intricate expression patterns of receptors for neuropeptides and neuromodulators.

Supplementary Figure 3 NPFR expressing neurons exhibit intricate expression of ion channels.

Supplementary Figure 4 Sequenced 750 bp of Ubc7 DNA displaying 18–23 bp deletion in 4 male flies harboring Vas-Cas9; Ubc7 gRNA. Yellow and purple colors represent gRNA1 and 2 complementary sequences. Underlined sequences represent the deleted sequences.
