# Supplementary material for: Transcriptome Analysis of NPFR Neurons Reveals a Connection Between Proteome Diversity and Social Behavior
Source: Front Behav Neurosci. 2021 Mar 31;15:628662. doi: 10.3389/fnbeh.2021.628662 (PMC8044454; doi:10.3389/fnbeh.2021.628662)
Supplement: Supplementary Figure 1 — List of behavioral features presented in Figures 5, 6. [file Image_1.pdf]

Definition	Description
Walk	Fly moves.
Stop	Fly is still.
Turn	Changes in fly’s direction.
Touch	Fly actively touches another fly.
Approach	Fly approaches another fly and perform interaction (active or passive).
Aggregation	Fly sits in a group of 3 or more flies.
Grooming	Fly grooms.
Chase	Fly chases another fly.
Chain	Chase with 3 or more flies.
Song	Fly moves one wing next to another fly.
Social clustering	Fly sits in a social cluster (aggregate)
Behavior bout length	Length of the longest sequence of frames in which the behavior occurred per fly.
absdtheta	Angular speed (rad/s).
velmag	Speed of the center of rotation (mm/s).
nflies_close	Number of flies within 2 body lengths (4a).
dcenter	Minimum distance from this animal’s center to other animal’s center (mm).
absthetadiff -anglesub	Absolute difference in orientation between current animal and closest animal based on anglesub (rad).
absthetadiff-nose2ell	Absolute difference in velocity direction between current animal and closest animal based on dnose2ell (rad).
Absphidiff-anglesub	Absolute difference in velocity direction between current animal and closest animal based on anglesub (rad).
absphidiff–nose2ell	Absolute difference in velocity direction between current animal and closest animal based on dnose2ell (rad).
anglefrom1to2-nose2ell	Angle to closest (based on distance from nose to ellipse) animal’s centroid in current animal’s coordinate system (rad).
angleonclosestfly	Angle of the current animal’s centroid in the closest (based on distance from nose to ellipse) animal’s coordinate system (rad).
