# Supplementary material for: Transcriptome Analysis of NPFR Neurons Reveals a Connection Between Proteome Diversity and Social Behavior
Source: Front Behav Neurosci. 2021 Mar 31;15:628662. doi: 10.3389/fnbeh.2021.628662 (PMC8044454; doi:10.3389/fnbeh.2021.628662)
Supplement: Supplementary Figure 2 — NPFR expressing neurons exhibit intricate expression patterns of receptors for neuropeptides and neuromodulators. [file Image_2.pdf]

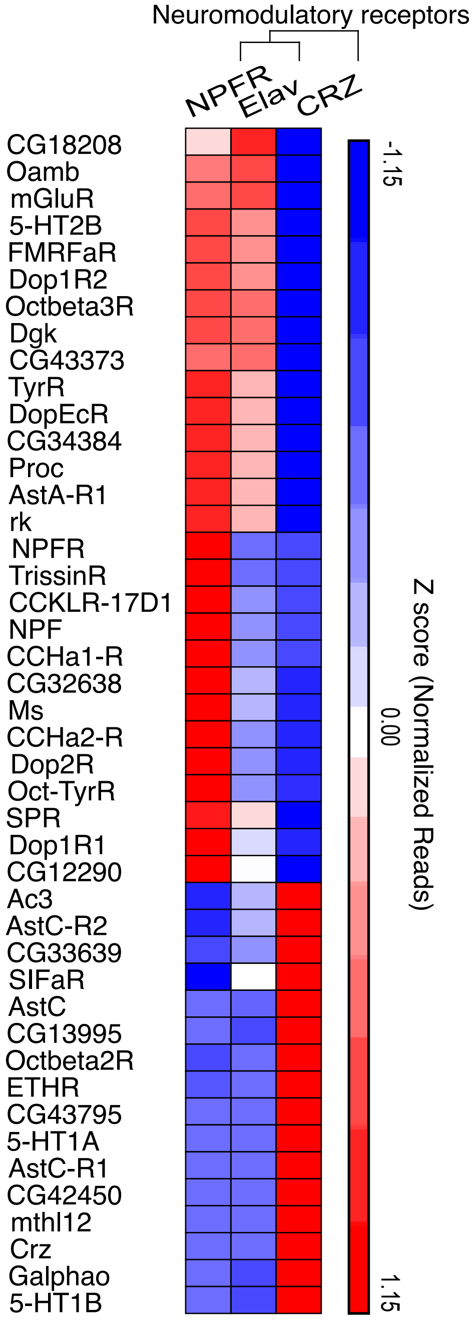

Neuromodulatory receptors
NPFR
Elav
CRZ
CG18208
Oamb
mGluR
5-HT2B
FMRFaR
Dop1R2
Octbeta3R
Dgk
CG43373
TyrR
DopEcR
CG34384
Proc
AstA-R1
rk
NPFR
TrissinR
CCKLR-17D1
NPF
CCHa1-R
CG32638
Ms
CCHa2-R
Dop2R
Oct-TyrR
SPR
Dop1R1
CG12290
Ac3
AstC-R2
CG33639
SIFaR
AstC
CG13995
Octbeta2R
ETHR
CG43795
5-HT1A
AstC-R1
CG42450
mthl12
Crz
Galphao
5-HT1B
-1.15
0.00
1.15
Z score (Normalized Reads)

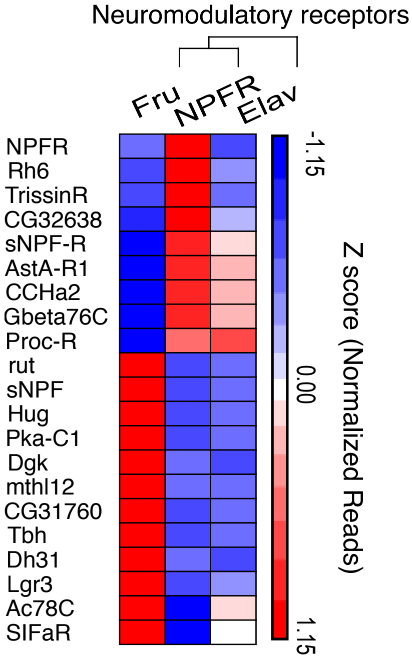

Neuromodulatory receptors
Fru
NPFR
Elav
NPFR
Rh6
TrissinR
CG32638
sNPF-R
AstA-R1
CCHa2
Gbeta76C
Proc-R
rut
sNPF
Hug
Pka-C1
Dgk
mthl12
CG31760
Tbh
Dh31
Lgr3
Ac78C
SIFaR
-1.15
0.00
1.15
Z score (Normalized Reads)

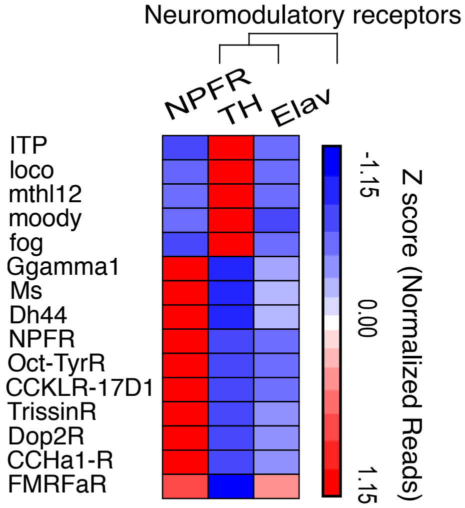

Neuromodulatory receptors
NPFR
TH
Elav
ITP
loco
mthl12
moody
fog
Ggamma1
Ms
Dh44
NPFR
Oct-TyrR
CCKLR-17D1
TrissinR
Dop2R
CCHa1-R
FMRFaR
-1.15
0.00
1.15
Z score (Normalized Reads)
