# Supplementary figures and images for: Transcriptome Analysis of NPFR Neurons Reveals a Connection Between Proteome Diversity and Social Behavior
Source: Front Behav Neurosci. 2021 Mar 31;15:628662. doi: 10.3389/fnbeh.2021.628662 (PMC8044454; doi:10.3389/fnbeh.2021.628662)

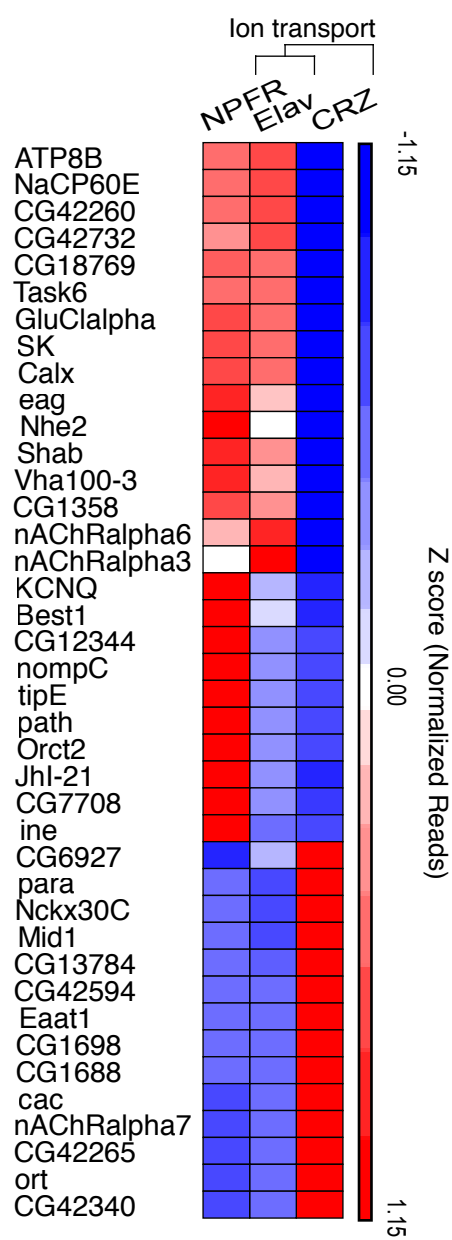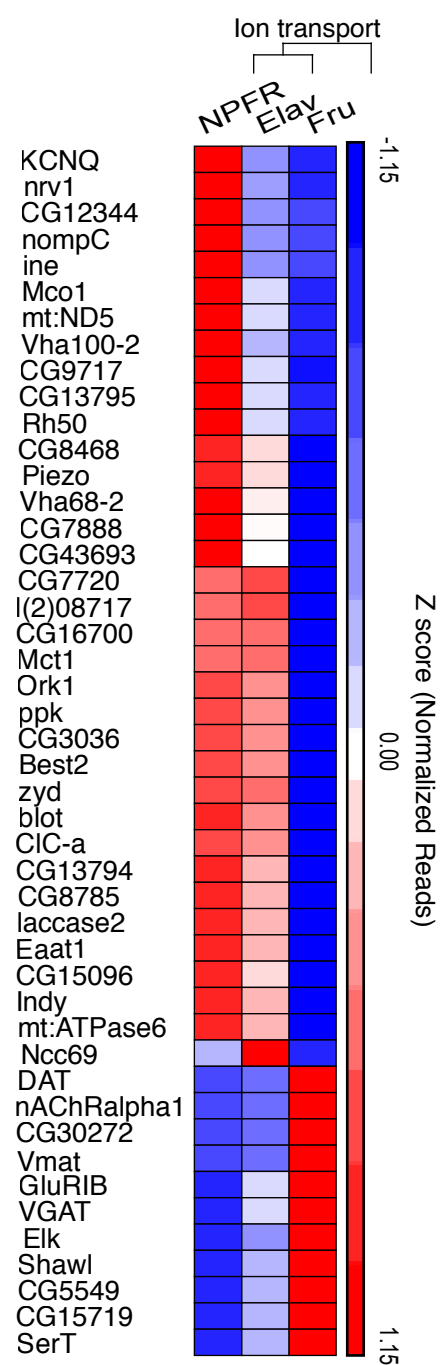

Supplement: Supplementary Figure 3 — NPFR expressing neurons exhibit intricate expression of ion channels. [file Image_3.pdf]
