# Supplementary material for: Transcriptome Analysis of NPFR Neurons Reveals a Connection Between Proteome Diversity and Social Behavior
Source: Front Behav Neurosci. 2021 Mar 31;15:628662. doi: 10.3389/fnbeh.2021.628662 (PMC8044454; doi:10.3389/fnbeh.2021.628662)
Supplement: Supplementary Figure 4 — Sequenced 750 bp of Ubc7 DNA displaying 18–23 bp deletion in 4 male flies harboring Vas-Cas9; Ubc7 gRNA. Yellow and purple colors represent gRNA1 and 2 complementary sequences. Underlined sequences represent the deleted sequences. [file Image_4.pdf]

Sample 1 - deletion of 20bp

AGAAAGCCACTCGATTCATTCGATATAAATAAACACGGAACACCGTTTGTGTTTATTATATTACGACGTGTCTGTATTTTGACCCGTGCCCGAGGAT
CTGGAAAGTTCGCGATTGCCGCCAAAACAAGCAAGGACCCCATTCGGGTGTAGCAGGTGCAGGAGCAGGTGGACAGGAAACGGAGAAGCAGCTGAAG
GAAACTCAAAGGAAGTGGTCACAGTGGGGAGAAGGAGCCCGTGAACTGAACCATCATCATTACCATCGAGCGCATTTAGGATGGCTGGGTCCGCACT
GCGCCGCCTGATGGCGGAATACAAACgtgagtcgaaagtccacagggcagcaacagctttcccttaccaccgctccgatcatctccagA**GTTAACAC**
TTGACCCGCCCGAGGGCATTGTGGCCG**GCCCCATCAGCGAGGACAAC**TTCTTCGAGTGGGAGGCACTGATTGCgtgagttaagatccccgaacgatc
gacacggatcggacagatgtggacagattaacccattaattgcactttgccctttgcagCGGACCTGAGGGCACTTGTTTCGAGGGCGGAGTGTTTC
CTGCCCGGCTCATCTTTCCGACCGACTATCCTCTGAGTCCGCCTAAAATGAAATTCACTTGTGACATGTTCCATCCCAACATATTCGCCGACGGGCG
GGTCTGCATATCAATACTACACGCACCCGGCGACGATCCCATGGGCTACGAGCTATCCGCGGAGCGCTGGAGTCCT**GTCCAGAGCGTGGAGAAGAT**

Sample 2 - Deletion of 21bp

AGAAAGCCACTCGATTCATTCGATATAAATAAACACGGAACACCGTTTGTGTTTATTATATTACGACGTGTCTGTATTTTGACCCGTGCCCGAGGAT
CTGGAAAGTTCGCGATTGCCGCCAAAACAAGCAAGGACCCCATTCGGGTGTAGCAGGTGCAGGAGCAGGTGGACAGGAAACGGAGAAGCAGCTGAAG
GAAACTCAAAGGAAGTGGTCACAGTGGGGAGAAGGAGCCCGTGAACTGAACCATCATCATTACCATCGAGCGCATTTAGGATGGCTGGGTCCGCACT
GCGCCGCCTGATGGCGGAATACAAACgtgagtcgaaagtccacagggcagcaacagctttcccttaccaccgctccgatcatctccagA**GTTAACAC**
TTGACCCGCCCGAGGGCATTGTGGCCG**GCCCCATCAGCGAGGACAAC**TTCTTCGAGTGGGAGGCACTGATTGCgtgagttaagatccccgaacgatc
gacacggatcggacagatgtggacagattaacccattaattgcactttgccctttgcagCGGACCTGAGGGCACTTGTTTCGAGGGCGGAGTGTTTC
CTGCCCGGCTCATCTTTCCGACCGACTATCCTCTGAGTCCGCCTAAAATGAAATTCACTTGTGACATGTTCCATCCCAACATATTCGCCGACGGGCG
GGTCTGCATATCAATACTACACGCACCCGGCGACGATCCCATGGGCTACGAGCTATCCGCGGAGCGCTGGAGTCCT**GTCCAGAGCGTGGAGAAGAT**

Sample 3 - Deletion of 23bp

AGAAAGCCACTCGATTCATTCGATATAAATAAACACGGAACACCGTTTGTGTTTATTATATTACGACGTGTCTGTATTTTGACCCGTGCCCGAGGAT
CTGGAAAGTTCGCGATTGCCGCCAAAACAAGCAAGGACCCCATTCGGGTGTAGCAGGTGCAGGAGCAGGTGGACAGGAAACGGAGAAGCAGCTGAAG
GAAACTCAAAGGAAGTGGTCACAGTGGGGAGAAGGAGCCCGTGAACTGAACCATCATCATTACCATCGAGCGCATTTAGGATGGCTGGGTCCGCACT
GCGCCGCCTGATGGCGGAATACAAACgtgagtcgaaagtccacagggcagcaacagctttcccttaccaccgctccgatcatctccagA**GTTAACAC**
TTGACCCGCCCGAGGGCATTGTGGCCG**GCCCCATCAGCGAGGACAAC**TTCTTCGAGTGGGAGGCACTGATTGCgtgagttaagatccccgaacgatc
gacacggatcggacagatgtggacagattaacccattaattgcactttgccctttgcagCGGACCTGAGGGCACTTGTTTCGAGGGCGGAGTGTTTC
CTGCCCGGCTCATCTTTCCGACCGACTATCCTCTGAGTCCGCCTAAAATGAAATTCACTTGTGACATGTTCCATCCCAACATATTCGCCGACGGGCG
GGTCTGCATATCAATACTACACGCACCCGGCGACGATCCCATGGGCTACGAGCTATCCGCGGAGCGCTGGAGTCCT**GTCCAGAGCGTGGAGAAGAT**

Sample 4– deletion of 18bp

AGAAAGCCACTCGATTCATTCGATATAAATAAACACGGAACACCGTTTGTGTTTATTATATTACGACGTGTCTGTATTTTGACCCGTGCCCGAGGAT
CTGGAAAGTTCGCGATTGCCGCCAAAACAAGCAAGGACCCCATTCGGGTGTAGCAGGTGCAGGAGCAGGTGGACAGGAAACGGAGAAGCAGCTGAAG
GAAACTCAAAGGAAGTGGTCACAGTGGGGAGAAGGAGCCCGTGAACTGAACCATCATCATTACCATCGAGCGCATTTAGGATGGCTGGGTCCGCACT
GCGCCGCCTGATGGCGGAATACAAACgtgagtcgaaagtccacagggcagcaacagctttcccttaccaccgctccgatcatctccagA**GTTAACAC**
TTGACCCGCCCGAGGGCATTGTGGCCG**GCCCCATCAGCGAGGACAAC**TTCTTCGAGTGGGAGGCACTGATTGCgtgagttaagatccccgaacgatc
gacacggatcggacagatgtggacagattaacccattaattgcactttgccctttgcagCGGACCTGAGGGCACTTGTTTCGAGGGCGGAGTGTTTC
CTGCCCGGCTCATCTTTCCGACCGACTATCCTCTGAGTCCGCCTAAAATGAAATTCACTTGTGACATGTTCCATCCCAACATATTCGCCGACGGGCG
GGTCTGCATATCAATACTACACGCACCCGGCGACGATCCCATGGGCTACGAGCTATCCGCGGAGCGCTGGAGTCCT**GTCCAGAGCGTGGAGAAGAT**

Figure S3: Sequenced 750 bp of Ubc7 DNA displaying 18-23 bp deletion in 4 male flies harboring Vas-Cas9; Ubc7 gRNA. Yellow and purple colors represent gRNA1 and 2 complementary sequences. Underlined sequences represent the deleted sequences.
